# Immediate versus early (24-hours) urinary catheter removal after elective minimally invasive colonic resection: study protocol for a randomized, multicenter, non-inferiority trial

**DOI:** 10.1186/s13063-022-06894-6

**Published:** 2022-11-22

**Authors:** Corrado Pedrazzani, Isacco Montroni, Cristian Conti, Giulia Turri, Caterina Foppa, Michele Carvello, Giovanni Taffurelli, Giampaolo Ugolini, Antonino Spinelli

**Affiliations:** 1 Division of General and Hepatobiliary Surgery, Department of Surgical Sciences, Dentistry, Gynecology and Pediatrics, University of Verona, Verona, Italy; 2 Division of Colon and Rectal Surgery, Faenza Hospital, Faenza, Italy; 3 Division of General Surgery, Ravenna Hospital, Ravenna, Italy; 4 Division of Colon and Rectal Surgery, IRCCS Humanitas Research Hospital, Rozzano, Milan, Italy; 5 Department of Biomedical Sciences, Humanitas University, Milan, Italy

**Keywords:** Colorectal surgery, Laparoscopy, ERAS, Urinary catheter

## Abstract

**Background:**

Enhanced Recovery After Surgery (ERAS) represents the standard of care in colorectal surgery. Among ERAS items, early removal of urinary catheter (UC) is considered a key issue, though adherence to this specific item still varies among centers. UC placement allows for monitoring of post-operative urinary output but relates to an increased risk of urinary tract infection (UTI), reduced mobility, and patient’s discomfort. Several studies investigated the role of early UC removal specifically looking at the rate of acute urinary retention (AUR) but most of them were retrospective, single-center, underpowered, cohort studies. The main purpose of this study is to compare the rate of AUR after immediate (at the end of the surgery) versus early (within 24 h from the completion of surgery) removal of UC in patients undergoing minimally invasive colonic resection (MICR). The secondary outcomes focus on goals that could be positively impacted by the immediate removal of the UC at the end of the surgery. In particular, the rate of UTIs, perception of pain, time-to-return of bowel and physical functions, postoperative complications, and length of hospital stay will be measured.

**Methods:**

This is a prospective, randomized, controlled, two-arm, multi-center, study comparing the rate of AUR after immediate versus early removal of UC in patients undergoing MICR. The investigators hypothesize that immediate UC removal is non-inferior to 24-h UC removal in terms of AUR rate. Randomization is at the patient level and participants are randomized 1:1 to remove their UC either immediately or within 24 h from the completion of surgery. Those eligible for inclusion were patients undergoing any MICR with an anastomosis above the peritoneal reflection. Those patients who need to continue urinary output monitoring after the surgery will be excluded. The number of patients calculated to be enrolled in each group is 108 based on an expected AUR rate of 3% for the 24-h UC removal group and considering acceptable an AUR of 9% for the immediate UC removal group.

**Discussion:**

The demonstration of a non-inferiority of immediate versus 24-h removal of UC would call into question the usefulness of urinary drainage in the setting of MICR.

**Trial registration:**

ClinicalTrials.gov NCT05249192. Prospectively registered on February 21, 2022.

**Supplementary Information:**

The online version contains supplementary material available at 10.1186/s13063-022-06894-6.

## Background

Enhanced Recovery After Surgery (ERAS) represents the standard of care in colorectal surgery mainly because of the proven impact in reducing postoperative morbidity and length of stay (LOS) [1, 2]. Among ERAS items, early removal of urinary catheter (UC) is considered a key item [3], although adherence to this specific item varies among centers [4, 5].

UC allows for continuous post-operative urinary output monitoring and prevents acute urinary retention (AUR) in the immediate postoperative period. On the opposite, UC is associated with an increased risk of urinary tract infection (UTI), reduced mobility, and patient discomfort [1, 6, 7].

Several studies investigated the role of early UC removal in colorectal surgery but cohorts were generally unbalanced, considering both rectal and colonic resections, open and laparoscopic approaches, and different analgesic regimens including epidural analgesia [8–10]. Besides, most of them were retrospective, single-center, cohort studies.

Li et al. retrospectively analyzed 110 patients submitted to open colonic resection for cancer within an ERAS program comparing 80 patients without any perioperative urinary catheter placement vs. 30 patients where UC was placed before the surgery. The AUR rate was 15% when no UC was positioned and 0% when intraoperative UC was used (*p* = 0.034) [11].

Althoff et al. presented their results from 4 groups of patients undergoing either open or minimally invasive colorectal surgery without catheter placement (27 patients), with catheter removed at the end of surgery (249 patients), UC removed within 24 h (214 patients), and more than 24 h after surgery (151 patients). No significant differences in AUR were observed among groups (3.7% vs. 6.8% vs. 4.2% vs. 2.6%; *p*=0.264). At the multivariate logistic regression, only pelvic surgery was found to be an independent risk factor for AUR (*p*=0.008). Patients with prolonged catheterization experienced a longer LOS (*p*=0.001) and a higher rate of UTI (*p*=0.017) [12].

Conti et al. recently published a retrospective series of 227 consecutive patients undergoing laparoscopic colonic resection analyzing the rate of AUR when UC was removed within 24 h vs. later in the hospital admission. Using a propensity score match, 76 patients with early (within 24 h from the completion of surgery) and 76 patients with standard (median, IQR: 2, 2–3 days) UC removal were compared demonstrating an analogous AUR rate (0% vs. 2.6%; *p* = 0.16). In the study group, patients that had their UC removed early also experienced better results in terms of postoperative complications (6.6% vs. 17.1%; *p* = 0.04), withdrawal of post-operative IV fluids (median, IQR: 1, 1–3 days vs. 2.5, 2–4 days; *p* < 0.001), time-to-return to independent walking (median, IQR: 1, 1–2 days vs. 2, 1–2 days; *p* = 0.02), time-to-tolerate oral feeding (median, IQR: 1.5, 1–2.75 days vs. 2, 1–3 days; *p* = 0.02), and LOS (median, IQR: 5, 4–7 days vs. 6, 5–7 days; *p* = 0.01) [13].

### Rationale for the trial

Despite the number of studies analyzing the feasibility and potential advantages of early UC removal after colorectal resection, current literature lacks strong evidence coming from randomized controlled studies with an adequate sample size. The goal of minimally invasive surgery is to minimize the stress of surgery along with the reduction of the number of unnecessary devices placed into patients’ bodies in the postoperative period. The demonstration of non-inferiority of immediate vs. early (within 24 h from surgery) UC removal would allow clinicians to remove another barrier moving towards a stress-free surgical experience for patients.

### Study aims and objectives

The primary aim of this study is to compare the rate of AUR after immediate compared to early (24-h) removal of UC in patients undergoing minimally invasive colonic resection (MICR). The study hypothesis is that immediate UC removal is non-inferior to 24-h UC removal in terms of AUR rate.

The secondary outcomes focus on items that could be positively impacted by the immediate removal of the UC at the end of the surgery. In particular, the rate of UTIs, perception of pain, time-to-return of bowel and physical functions, postoperative complications, and LOS will all be measured.

### Trial design

This is a prospective, randomized, controlled, two-arm, multi-center study evaluating the feasibility of immediate versus early (within 24 h from the completion of surgery) UC removal in the context of MICR. Randomization is at the patient level and participants are randomized 1:1 to undergo immediate *versus* 24-h UC removal.

The protocol purpose is to demonstrate the non-inferiority of immediate compared to 24-h UC removal in terms of AUR. We assume that immediate UC removal is not significantly inferior to 24-h UC removal. Based on the current literature, to test the alternative hypothesis of non-inferiority of immediate UC removal versus 24-h UC removal, the sample size is calculated using the incidence of AUR. Available data show that the AUR rate can be estimated as high as 3% after MICR [14–16].

**Fig. 1:**
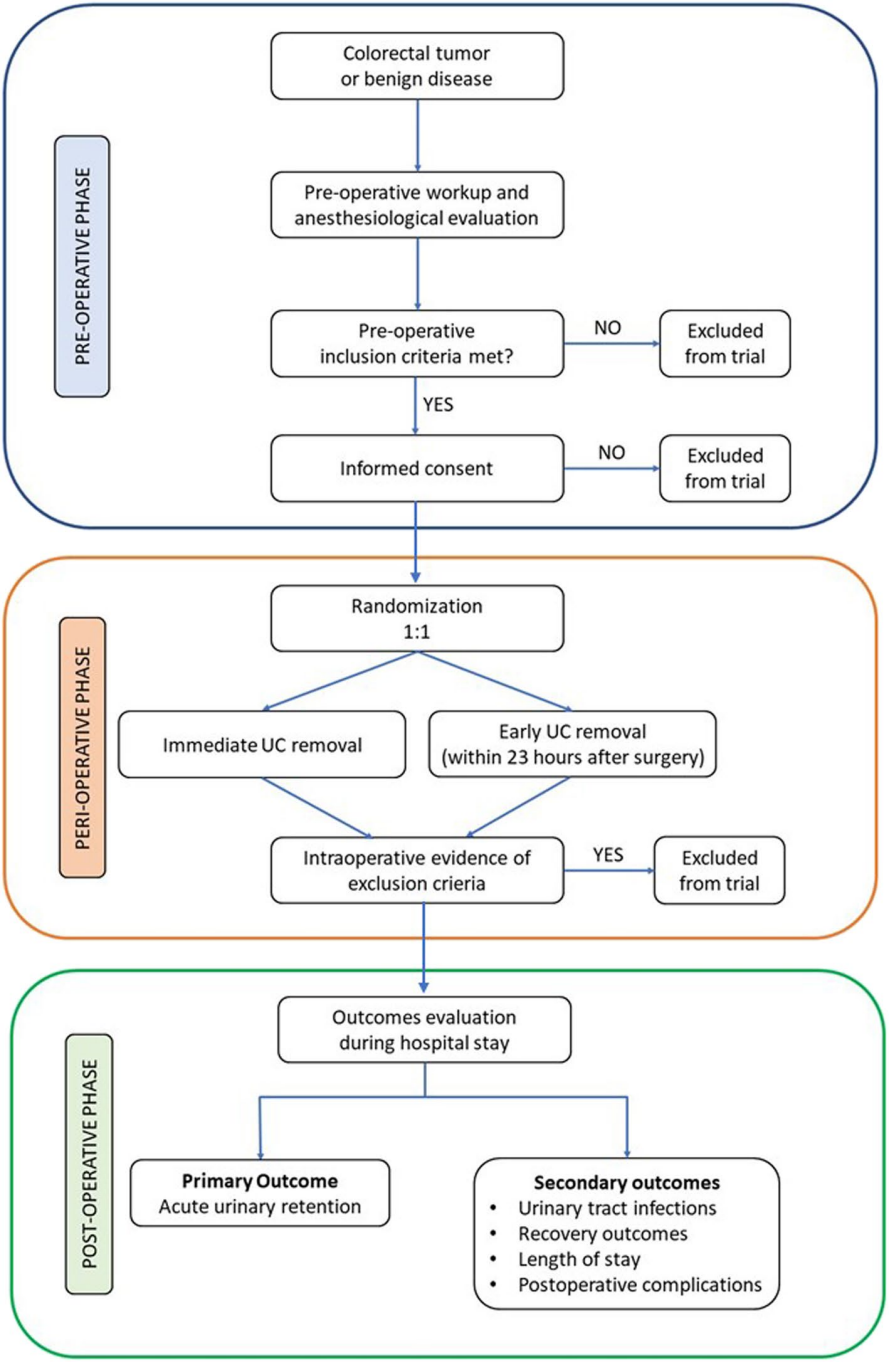
Overview of the trial design

**Fig. 2:**
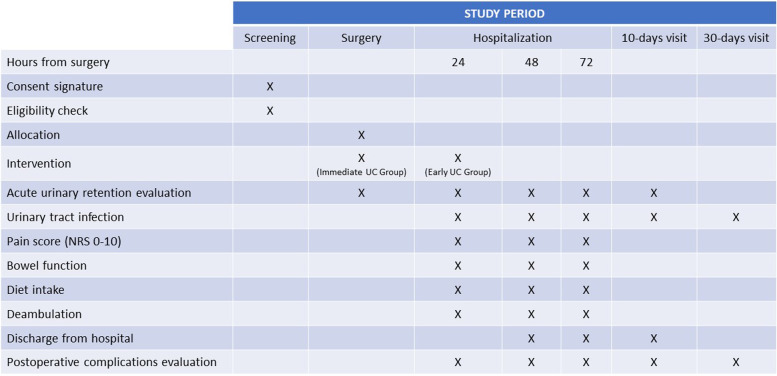
Schedule of enrolment, interventions, and assessments. POD, postoperative day; AUR, acute urinary retention; UTI, urinary tract infection; NRS, numerical rating scale

Occurrence of AUR, need for postoperative UC placement independently from the cause (e.g., AUR, need of re-operative surgery, and need of admission to the intensive care unit), and UTI will be recorded for every patient in the study., Patients will be asked to report about pain according to a numerical rating scale (NRS) at 6, 12, 24, 48, and 72 h after surgery. Return to passive and active mobilization, return to bowel function as well as the ability to tolerate oral diet will be also recorded together with the occurrence of postoperative complications and the length of hospital stay. The study flow diagram and time of collection of outcomes are shown in Figs. 1 and 2.

This study is reported in accordance with the Standard Protocol Items: Recommendations for Interventional Trials (SPIRIT) Checklist for clinical trial protocols (Additional file 1).

### Setting

Participants will be recruited and operated on at the Division of General and Hepatobiliary Surgery, University of Verona Hospital Trust; Division of Colon and Rectal Surgery, Humanitas Clinical and Research Hospital; Division of General Surgery, Ravenna Hospital; and the Division of Colon and Rectal Surgery, Faenza Hospital AUSL Romagna. The caseload of colorectal resections for each center is greater than 150 cases per year.

### Study duration

Recruitment of patients will start in February 2022. The planned duration of the study is 16 months.

### Participants

Patients scheduled to undergo elective MICR.

### Inclusion criteria

1. Age between 18 years and 80 years old
2. Patients scheduled to undergo minimally invasive resection of the colon under general anesthesia
3. Willingness to participate
4. Compliance to study purpose
5. Written informed consent

### Exclusion criteria

1. Need for an anastomosis below the anterior peritoneal reflection
2. Need for a major resection other than colorectal
3. Need for post-operative intensive care monitoring or intensive care unit (ICU) stay
4. Anesthesia time longer than 300 min
5. Presence of chronic indwelling UC
6. Presence of an entero-vesical fistula
7. Need for ureteral stent placement, bladder resection, or repair
8. A previous and unsolved history of AUR or overt voiding dysfunction

### Interventions

#### Anesthesia protocol

General anesthesia will be conducted according to each center protocol following ERAS guidelines [2]. Deep curarization and goal-directed therapy will be adopted in all centers. No spinal or epidural analgesia will be allowed; conversely, transversus abdominis plane (TAP) bloc or local wound infiltration are encouraged. Duration of anesthesia, analgesia regimen and total amount of IV fluids will be registered.

#### Surgical technique

Multiport laparoscopic or robotic surgery as well as single incision laparoscopic surgery (SILS), or natural orifice specimen extraction surgery (NOSES) will be allowed. Site of minilaparotomy for specimen extraction will be decided by each operating surgeon according to her/his usual technique. Similarly, an intra-corporeal or extra-corporeal anastomosis will be performed according to surgeon’s preference. Conversion is defined as the inability to complete all intended steps using a minimally invasive approach. Duration of surgery, blood loss, adopted surgical technique, need for conversion, and site and length of minilaparotomy will be registered.

#### Interventional treatment

Single IV dose of prophylactic antibiotics within 1 h from skin incision will be administered according to local protocols. Foley catheter (14-16F) will be positioned upon induction using an aseptic technique. At the end of surgery, a member of the surgical staff not involved in the operation will instruct the operating surgeon on the timing of UC removal, according to the group of intervention that the subject was assigned. No prophylactic use of medications to prevent acute urinary retention (e.g., Alpha-lithic agents) will be allowed in both groups.

#### Postoperative analgesia and PONV prophylaxis

Intravenous acetaminophen 1000 mg will be administered 3 times a day (every 8 h) from the day of surgery until post-operative day (POD) 3. Ketorolac 30 mg or tramadol 100 mg will be prescribed as rescue analgesics. PONV prophylaxis is based on the intravenous administration of Metoclopramide 10 mg every 8 h from the day of surgery until POD 3. According to ERAS guidelines, post-operative IV fluids should be discontinued on POD1. Any reason for IV fluids administration after POD1 will be recorded together with its amount.

#### Withdrawal

All patients are freely informed to participate in this study and can decide to withdraw from this trial at any time. Any information regarding patients who decide to withdraw from the protocol are excluded from the final analysis.

### Outcome measures

#### Primary endpoint

To demonstrate the non-inferiority of immediate compared to early (within 24 h from the completion of surgery) removal of UC in patients undergoing MICR in terms of AUR. AUR is defined as the inability to urinate within 8 h after UC removal or difficulty of voiding with a postvoid residual volume (PVR) measured by ultrasound greater than 300mL.

#### Secondary outcome measure

To demonstrate the non-inferiority of immediate compared to 24-h removal of UC for the other short-term postoperative outcomes that could be influenced by the timing of UC removal:

1. Rate of UTI defined by the presence of symptoms (dysuria, frequency, urgency, suprapubic pain, hematuria, or testicular pain) and positive urine culture. Urine culture is considered positive when yielding more than 10^5^ colony-forming units per ml.
2. Rate of UC reinsertion considering all causes (e.g., AUR as well as need for redo surgery, need for ICU admission, etc.).
3. Pain control until POD 3. Intensity of pain is evaluated according to the NRS score and measured at 6, 12, 24, 48, and 72 h from surgery.
4. Return to bowel function. Return to bowel function is evaluated considering the time (days) needed for the bowel to open to gas and stool as well as for the patient to tolerate a liquid and solid diet.
5. Return to passive and active mobilization. Mobilization is evaluated considering the time (days) needed for sitting on a chair and walking.
6. Postoperative morbidity. Postoperative morbidity is classified according to the Clavien-Dindo classification. Overall, general, and surgery-related postoperative complications as well as need for readmission occurring within 30 days after surgery will be considered.
7. Possibility to be discharged from the hospital. Ability to go back home is computed considering postoperative length of hospital stay (days).

### Sample size calculation

The null hypothesis is that the immediate UC removal is significantly inferior to the 24-h UC removal. Based on the current literature, in order to test the alternative hypothesis of non-inferiority of immediate UC removal vs. 24-h UC removal, the sample size is calculated using the incidence of AUR. Available data show that the AUR rate can be estimated as high as 3% after MICR [14–16].

Sample size was calculated based on the following data. Considering an expected AUR rate of 3% (p1) for the 24-h UC removal group, we choose the non-inferiority limit of δ = 6% with δ = p2-p1, considering acceptable a p2 of 9% for the immediate UC removal group. We calculated that 200 patients are required in each group to give 80% power at a 5% significance level. Considering 8% dropout, the total number of patients to be enrolled is 216, 108 per group (Non-Inferiority Tests for the Difference Between Two Proportions, PASS 14)

### Allocation and sequence generation

Patients satisfying the inclusion criteria and willing to enter the study are randomized to one of the two study arms after signing the informed consent. The randomization is performed in a 1:1 fashion. The allocation to immediate or early UC removal group is achieved by creating a randomization list. The randomization list has been obtained by an online program available at https://www.sealedenvelope.com/simple-randomiser/v1/lists. A common list is created for all participating centers and patients’ allocation communicated by a member of the proposing center to a member of other centers the morning of surgery. The operating surgeon will be blinded to allocation of the patients until the end of surgery.

### Data collection and management

Trained members from each surgical staff oversee the data collection and recording. Baseline demographic data, as well as preoperative, intraoperative, and postoperative variables, are collected using a specifically created datasheet and stored in a specifically created dataset. The same datasheet and dataset are used in all centers. Data on patients’ comorbidities were registered with particular interest to previous prostatic surgery, history of prostatic disease, or medical therapy for benign prostatic hyperplasia.

To reduce as much as possible the variability among patients in reporting symptoms and pain intensity, the patient is instructed on symptoms and NRS scale by the same member of the surgical staff at the time of accrual (15–20 days before surgery) and the day before surgery. To reduce the variability related to data collection, the same member will collect all other investigated variables.

### Statistical analysis

Statistical analyses will be performed on an intention-to-treat (ITT) basis using IBM® SPSS® Statistics software version 21.0 (IBM Corporation, Armonk, NY).

An intention-to-treat analysis (ITT) will be used on all randomized subjects and, where appropriate, an imputation method will be established. It will be also performed per-protocol (PP) analysis that will include all subjects who have finished the catheter removal without major deviations from the research protocol. In non-inferiority studies, both ITT and PP analyses are recommended and should support non-inferiority.

The primary endpoint is AUR. Non-inferiority in the main outcome of the immediate UC removal group compared to 24-h UC removal will be evaluated by test for proportions. The non-inferiority of the immediate UC removal group will be accepted if the upper limit of the 95% confidence interval of the difference in proportions is greater than the non-inferiority threshold value of 6%.

The secondary objectives are assessing the benefits in choosing one procedure or the other, immediate UC removal versus 24-h UC removal. Descriptive statistics and plots will be used to compare the two groups taking into account: (1) UTI rate; (2) overall rate of re-catheterization; (3) pain intensity at 6, 12, 24, 48, and 72 h after surgery; (4) time from surgery to resumption of first oral intake (liquid and soft diet), bowel movements (flatus and defecation), mobilization to chair, and walking; (5) rate of overall, general, and surgery-related post-operative complications; (6) length of hospital stay.

### Auditing trial conduct plan

There are no plans for trial audit given the short duration of the trial intervention and follow-up.

### Dissemination plans

The results of the trial will be published in peer-reviewed academic journals and presented at national and international scientific meetings. Additionally, a lay summary containing the study aim, salient findings, conclusions, and a take home message will be prepared and distributed through electronic media including newsletters, social media, and websites.

## Discussion

UC is routinely adopted after major surgery for preventing AUR and facilitating fluid balance monitoring. Nonetheless, the presence of a UC is associated with reduced mobility and increased patients’ discomfort [1]. Additionally, after colorectal surgery, urinary catheter placement could be complicated by a UTI rate of almost 4%, which represents the highest rate among gastrointestinal surgical procedures [6, 17]. Although recommended by ERAS guidelines [2], early UC removal is one of the most problematic items to be achieved. In a recent paper, the PeriOperative Italian Society (POIS) showed a rate of UC removal within 24 h from surgery of 72.5% among patients undergoing an elective colorectal resection within an ERAS program [3]. Nonetheless, compliance to this item has been shown to increase up to 96% when specific protocols are implemented [14].

Early UC removal together with early withdrawal of IV fluids have been reported to be crucial for reducing overall morbidity and shortening LOS [3]. In fact, it is well demonstrated that IV fluid over prescription is associated with cardiopulmonary overload and intestinal and anastomotic edema [18, 19].

Although feasibility and advantages of early UC removal following colorectal surgery have been demonstrated, the time extent of UC to be in place remains debated [11–13, 20]. Current evidence is based on retrospective, unpowered and unbalanced cohort studies. Mixing colon and rectal surgery, open and minimally invasive approach, epidural as well as strong opioid-based analgesia regimens are among the most common bias making a definitive statement difficult to be made.

In this randomized controlled trial, we will focus on MICRs not requiring pelvic dissection. This will allow us to analyze a homogeneous cohort of patients in terms of the extent of surgery and pain management protocols. Epidural anesthesia is no longer suggested by ERAS guidelines in laparoscopic surgery and, being a well-known risk factor for AUR, it has been considered an exclusion criterion [2]. This together with the fact that, at participating centers, open surgery is mostly reserved to urgent or very advanced cases helps the results to be focused on patients undergoing MICRs specifically.

Even though completely avoiding UC placement could be considered an option [12, 20], evidence about this approach is currently even poorer from the literature. In addition, the frequent use of suprapubic incision for specimen extraction, both in left- and right-sided colonic resections, lead us to prefer the current study approach.

In conclusion, this study comparing immediate versus early (within 24 h from surgery) UC removal represents a solid option to demonstrate the possibility of moving a step forward in reducing the stress and discomfort for our patients undergoing elective minimally invasive colonic surgery.

### Trial status

Recruitment has started on February 15, 2022, and it will be completed within the end of June 2023 (UniVR Prog. 3589CESC).

## Supplementary Information

**Additional file 1.** Standard Protocol Items: Recommendations for Interventional Trials (SPIRIT) checklist.

## Data Availability

The datasets used and/or analyzed during the current study are available from the corresponding author (CP) on reasonable request.

## Abbreviations

AUR: Acute urinary retention
ERAS: Enhanced Recovery After Surgery
ICU: Intensive care unit
IQR: Interquartile range
ITT: Intention-to-treat
IV: Intravenous
LOS: Length of stay
MICR: Minimally invasive colonic resection
NOSES: Natural orifice specimen extraction surgery
NRS: Numerical rating scale
POD: Post-operative day
PP: Per-protocol
PVR: Postvoid residual volume
TAP: Transversus abdominis plane
UC: Urinary catheter
UTI: Urinary tract infection
